# Supplementary material for: A Review of the Respiratory Health Burden Attributable to Short-Term Exposure to Pollen
Source: Int J Environ Res Public Health. 2022 Jun 20;19(12):7541. doi: 10.3390/ijerph19127541 (PMC9224249; doi:10.3390/ijerph19127541)
Supplement: Supplementary file 1 [file ijerph-19-07541-s001.zip › ijerph-1724659-supplementary.pdf]

Supplementary Material

Table S1. Search strategy to identify relevant papers on PubMed database.

Type of articles	Outcomes	Search terms
Systematic reviews (inception-2022)	Any of the respiratory outcomes of interest	(pollen) AND ("systematic review")
Original articles (2020-2022)	Airway inflammation	((pollen) AND ("airway inflammation")) AND (("2020/01/01"[Date - Publication] : "3000"[Date - Publication]))
Original articles (2020-2022)	Thunderstorm asthma	((pollen) AND ("thunderstorm asthma")) AND (("2020/01/01"[Date - Publication] : "3000"[Date - Publication]))
Original articles (2020-2022)	Asthma in children	(((pollen) AND (asthma)) AND (child*)) AND (("2020/01/01"[Date - Publication] : "3000"[Date - Publication]))
		(((pollen) AND (asthma)) AND (paed*)) AND (("2020/01/01"[Date - Publication] : "3000"[Date - Publication]))
Original articles (inception -2022)	Asthma in adults	(((pollen) AND (asthma)) AND (adult)) AND (English[Language])
Original articles (inception -2022)	COPD	(pollen) AND (COPD)
Original articles (inception -2022)	General Practice	(pollen) AND (“general practice”)
Original articles (2020-2022)	Upper and lower respiratory symptoms	((pollen) AND ("respiratory symptoms")) AND (("2020/01/01"[Date - Publication] : "3000"[Date - Publication]))
Original articles (2020-2022)	Lung function	((pollen) AND ("lung function")) AND (("2020/01/01"[Date - Publication] : "3000"[Date - Publication]))

Original articles	Interactions	No search terms were used. Effect modifiers were determined from relevant papers identified from the search.
